# Current Understanding of the Applications of Photocrosslinked Hydrogels in Biomedical Engineering

**DOI:** 10.3390/gels8040216

**Published:** 2022-04-01

**Authors:** Juan Liu, Chunyu Su, Yutong Chen, Shujing Tian, Chunxiu Lu, Wei Huang, Qizhuang Lv

**Affiliations:** 1 College of Biology & Pharmacy, Yulin Normal University, Yulin 537000, China; liujuan5269@163.com (J.L.); suchunyu10@163.com (C.S.); c2922095838@163.com (Y.C.); tian011028@163.com (S.T.); lu18934908250@163.com (C.L.); 2 Guangxi Key Laboratory of Agricultural Resources Chemistry and Biotechnology, Yulin 537000, China

**Keywords:** water gel, photocrosslinking, synthetic polymer, natural polymer modification, biomedical-engineering applications

## Abstract

Hydrogel materials have great application value in biomedical engineering. Among them, photocrosslinked hydrogels have attracted much attention due to their variety and simple convenient preparation methods. Here, we provide a systematic review of the biomedical-engineering applications of photocrosslinked hydrogels. First, we introduce the types of photocrosslinked hydrogel monomers, and the methods for preparation of photocrosslinked hydrogels with different morphologies are summarized. Subsequently, various biomedical applications of photocrosslinked hydrogels are reviewed. Finally, some shortcomings and development directions for photocrosslinked hydrogels are considered and proposed. This paper is designed to give researchers in related fields a systematic understanding of photocrosslinked hydrogels and provide inspiration to seek new development directions for studies of photocrosslinked hydrogels or related materials.

## 1. Introduction

Hydrogels are crosslinked networks of polymers. They have excellent hydrophilicity and can absorb large amounts of water or tissue fluid. At the same time, their volumes can expand to thousands of times that of the anhydrous state [1]. Water absorption and water retention of hydrogels are closely related to the molecular structure of crosslinking network, hydrophilicity, and crosslinking degree of monomers. If the crosslinking network structure is too tight, its water absorption will be reduced. The higher the hydrophilicity of the monomer structure, the better the hydroscopicity. Too high a crosslinking degree will lead to a dense structure of hydrogel, thus reducing water absorption. When the water content is within a certain range, hydrogels have softness and a rubbery consistency similar to that of living tissue, which demonstrates their excellent biocompatibility for cells and tissues [2]. Therefore, hydrogels have great application value in biomedical engineering.

In hydrogels, crosslinking is the key to avoiding dissolution of hydrophilic-polymer chains or segments. Hydrogels can be divided into physical and chemical hydrogels according to differences in crosslinking modes between polymers. In physically crosslinked hydrogels, polymers usually form three-dimensional network structures through hydrogen bonding, ionic bonding, hydrophobic bonding, chain entanglement, microcrystal formations, electrostatic interactions, etc. [3]. Such crosslinking is relatively simple and convenient, but the resulting network structure is not uniform, the mechanical strength is poor, and the crosslinking is usually reversible. Physical gels can subsequently be degraded by changes in temperature, pH, or ionic strength in the environment. This limits their use in complex internal environments. In contrast, chemical crosslinking that usually occurs by covalent bonds is usually irreversible and thus stable under changing conditions. In addition, hydrogels obtained by chemical crosslinking generally have better mechanical stability [4]. Therefore, chemically crosslinked hydrogels are more common in biomedical applications.

There are many methods for chemically crosslinking hydrogels, such as crosslinking polymerization of complementary functional groups, enzyme-induced polymerization, photo- or heat-induced free-radical polymerization, and high-energy irradiation-crosslinking polymerization [5]. Among them, the conditions of crosslinking polymerization with complementary functional groups and enzyme-induced polymerization are mild, and unnecessary functional molecules are not introduced, so they are the two preferred crosslinking methods [6,7]. However, in practical applications, the small molecules and macromolecules suitable for these two crosslinking methods are relatively limited, so there are few hydrogels prepared by using these two polymerization methods [8]. In contrast, light- or heat-induced radical polymerization is a common method in chemical crosslinking. In these methods, initiators must be added to a solution of crosslinked molecules to induce cracking into free radicals with light or heat, attacking the polymer chain withthe crosslinking molecules and initiating a chain reaction to complete polymerization [9]. Therefore, photo- or heat-induced free-radical polymerization is especially widely used because of its simplicity and speed.

With the development of cross-disciplines and deepening of scientific research, an increasing number of photocrosslinked hydrogels have been developed and applied in biomedical engineering. It is therefore necessary to give a summary and a review of the subject, as well as a glimpse into possible future development prospects. First, we introduce the compositions of photocrosslinked hydrogels, including synthetic monomers and modified monomers based on natural materials. Subsequently, we summarize hydrogels with different morphologies, such as films, fibers, microspheres, microneedles, and amorphous (injectable) hydrogels based on these monomers. Then, we summarize recent applications of these hydrogels in biomedical engineering. Finally, based on current development status, we put forward views on the development prospects forphotocrosslinked hydrogels in biomedical engineering. Overall, we hope that this review will give researchers a better understanding of this field and promote further development.

## 2. Chemically Synthesized Molecules for Preparation of Photocrosslinked Hydrogels

To prepare photocrosslinked hydrogels exhibitinggood biocompatibility, many polymerizable small molecules have been synthesized. These small molecules usually have carbon–carbon unsaturated bonds, and the polymer network is formed by bond breaking and addition reaction under initiator and light. Depending on the type of polymerizable group, these molecules can be roughly divided into four classes: ethylene, acrylic, acrylamide, and acrylate (Table 1).

### 2.1. Ethylenes

Structurally, ethylene is the simplest functional group among polymerizable molecules. Photopolymerization of ethylene molecules was also the earliest method discovered and applied. In early studies, styrene and other monomers were mainly used to synthesize resins due to their poor water solubility, and they were usually polymerized by high-energy radiation or heat [10]. At present, thevinyl molecules used in hydrogel preparations are mainly N-vinyl pyrrolidone (NVP). NVP itself has low viscosity, high reactivity, and limitedskin irritation [11], so it is widely used as a typical ethylene molecule [12]. Kao et al. first reported NVP photocrosslinking with four different comonomers to prepare a series of UV-curable bioadhesives with a high water uptake ranging from 25 to 350 wt% [13]. Subsequently, Fechine et al. studied the effects of crosslinking NVP with other polymerizable molecules such as hydrogen peroxide [14,15]. Lee and Devine et al. provideda detailed discussion on the network structure of copolymerized NVP and polyethylene glycol diacrylate (PEGDA) hydrogels and found that the molecular weight of the main chain was mainly related to the NVP content and was not affected by polymerization time [16,17]. In addition to NVP, a variety of other ethylene molecules have been reported for photocrosslinking polymerizations. For example, Sahiner et al. successfully prepared photocrosslinked bulk polyethylene phosphonic acid (PVPA) by mixing and crosslinking PEGDAs with different molecular weights [18,19]. Ren et al. synthesized a hydrogen-bonded calcium-crosslinked PVDT-PAA hydrogel from 2-vinyl-4,6-diamino-1,3,5-triazine (VDT), acrylic acid (AA), and PEGDA through one-step photopolymerization [20]. However, due to the restriction of water solubility and reactivity, vinyl monomers are still rarely used in the preparation of photocrosslinked hydrogels [21,22].

### 2.2. Acrylic Acid

Acrylic-acid (AA) molecules have photoactive groups and good water solubility, and due to the free carboxyl structure remaining after polymerization, theycan swell or shrink with environmental pH changes, electric fields, enzyme reactions, and temperature, so that the hydrogel exhibits a tunableresponse. In addition, these free carboxyl groups can interact with other groups under certain conditions, so the hydrogel network can be functionalized by reactions with carboxyl groups or interactions with solutes [23,24,25,26]. Therefore, photocrosslinked hydrogels based on AA molecules are very common and are often used for biological adsorption and environmental purification. For example, Liu et al. successfully synthesized a b-cyclodextrin or polyacrylic-acid nanocomposite (b-CD/PAA/GO) grafted with graphene oxide (GO) based on polyacrylic acid (PAA) through an esterification reaction, and prepared a composite PAA hydrogel, with an adsorption capacity of up to 248 mg/g fordye molecules in wastewater [27]. Hu et al. prepared a new PAA hydrogel by improving and optimizing the crosslinking agent usedin the AA polymerization process, and the adsorption capacity fordye molecules reached a record-breaking 2100 mg/g under neutral conditions [28]. Ma and Kong et al. combined PAA hydrogels with organic montmorillonite or GO, and the resultingcomposite hydrogel showed good adsorption capacitiesfor lead ions (Pb^2+^) with an adsorption capacity of 223.84 mg/g [29] and cadmium ions (Cd^2+^) with maximum adsorption capacity up to 316.4 mg/g [30]. In addition, PAA hydrogels can be used in the construction of flexible devices due to their good biocompatibility and flexibility. Based on this, Lu et al. combined PAA with nanocellulose to prepare a flexible hydrogel that can be used for skin sensing [31]. Clearly photocrosslinked hydrogels based on AA derivatives show a wide range of applications.

### 2.3. Acrylamide

Unlike liquid AA, acrylamide (AAm) molecules are usually solid and exhibit good water solubility. The AAm molecule does not have a free carboxyl group, but instead has a neutral amide bond, so polyacrylamide (PAAm) hydrogels do not exhibit pH responsiveness but show an equilibrium water content in the range of 94.73–96.26 wt%. Thanks to this, PAAm hydrogels maintain stability in environments with variable solutes [32]. However, the amide bond can also be partially hydrolyzed into a carboxyl group under certain conditions, and the PAAm hydrogel can function as a partial PAA hydrogel. For example, Wang et al. used this principle to prepare enzyme-functionalized microspheres for detection and cleaning of objects [33].

If a hydrophobic isopropyl group is introduced to the other end of the acrylamide molecule, isopropyl acrylamide (NIPAm) is obtained. After photocrosslinking, the resulting polyisopropyl acrylamide (PNIPAm) has a critical phase-transition temperature. When the temperature of the PNIPAm hydrogel is increased above the critical phase-transition temperature, the volume of the hydrogel shrinks significantly, and vice versa. It is worth mentioning that the critical phase-transition temperature of PNIPAm hydrogels is 32 °C and its low critical-dissolution temperature is close to the physiological temperature, it has important application value in biomedical engineering [34]. Zhang et al. prepared PNIPAm-hydrogel microspheres loaded with drugs, which shrank at physiological temperatures and released the loaded drugs for wound repair and disease treatment [35]. When PNIPAm hydrogel is combined with a substance susceptible to photothermal conversions, the new material exhibits photoresponsiveness, which can be used to deter counterfeiting [36,37].

### 2.4. Acrylates

The reaction of an acrylic derivative (acrylic or methacrylic acid) with the terminal hydroxyl group of another molecule yields an acrylate that can be used for photocrosslinking. For example, polyethylene glycol acrylate (PEGMA), polyethylene glycol diacrylate (PEGDA), polyethylene glycol dimethacrylate (PEGDMA) and other molecules that were obtained after modification at the end of polyethylene glycol (PEG) can be photocrosslinked to form the corresponding polymer network. Compared with acrylic acid and acrylamide hydrogels, acrylate hydrogels are not sensitive to changes in environmental temperature, pH, or other conditions, and are not convenient for functional-group modification; when the surrounding environment changes, this kind of hydrogel maintains good stability. In addition, some acrylate hydrogels, such as PEGDA hydrogels, show good water absorption (about 60 wt%), good biocompatibility, and good adhesion resistance [38]. Therefore, this kind of hydrogel can be used in biomedicine to construct a stable hydrogel skeleton for direct contact with cells or human tissues, biological analyses, and other applications [39]. Hou et al. prepared photocrosslinkedfibers composed of PEGDA with different molecular weights and studied the conversion of acrylate bonds in the hydrogel and the mechanical properties of the fibers in detail. Hou et al. prepared a hydrogel microsphere based on a PEGDA hydrogel and realized the detection of glycoprotein molecules in the solution [40].

## 3. Chemically Modified Natural Materials

Compared with synthetic materials, natural materials exist widely in nature and have incomparable advantages in sourcing and storage. In addition, natural materials usually have good biocompatibility; they are safer than synthetic materials for use in the biomedical field. At present, hydrogels prepared from natural materials play important roles in cell culture, tissue engineering, and other fields. However, natural materials usually do not have photocrosslinkable groups. In addition to a few complementary functional-group reactions, crosslinking based on natural materials usually relies on physical crosslinking. In practice, the mechanical strength and stability are poor. Therefore, researchers have proposed various strategies for modifying natural materials. The introduction of photocrosslinking groups makes the preparation of natural material hydrogels simpler and more convenient, solves the problems of mechanical strength and poor stability, and further expands their applications. Natural materials used for modification and preparation of crosslinked hydrogels are mainly divided into two categories depending on their composition: polysaccharides and proteins or peptides.

### 3.1. Polysaccharides

Polysaccharides are carbohydrates with complex and large molecular structures formed by dehydration and condensation of multiple monosaccharide molecules. They are widely distributed in nature and play important roles [41]. For example, peptidoglycan and cellulose are components of the cytoskeletal structure of plants and animals, and starch and glycogen are important energy-storage materials used by animals and plants. The monosaccharides that make up various polysaccharides often have free functional groups; there are many active groups on the polysaccharide chain that can be modified toallow photocrosslinking. Common modified crosslinked hydrogels include alginate, hyaluronic acid, chitosan, heparin, chondroitin sulfate, gellan gum, cyclodextrin, and dextran, among others.

**Figure 1:**
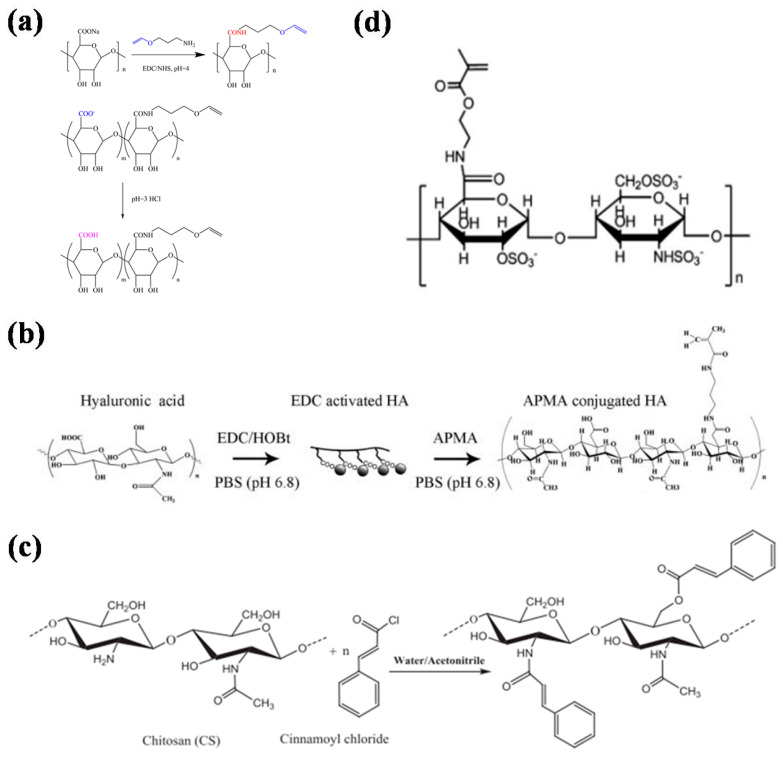
Synthetic route map or structural formula of the photocrosslinked hydrogel. (**a**) Synthetic route for aminopropyl vinyl ether-modified alginic acid [45]. (**b**) Synthetic route to N-(3-aminopropyl) methylacrylamide-modified hyaluronic acid [49]. (**c**) Synthetic roadmap for cinnamoyl chloride chloride-modified [51]. (**d**) Structural formula of heparin modified by methyl acrylate [52].

Alginate is a natural polysaccharide that can form a physical hydrogel by chelating its independent hydroxyl group with divalent and trivalent metal and heavy-metal ions. It has wide application value for drug delivery, wound repair, and tissue engineering [42,43]. However, physically crosslinked alginate hydrogels have limitations in practical applications due to their uncontrollable gluing speed and instability after gelation. Therefore, many researchers have modified alginate to graft photocrosslinking groups, and realize chemical crosslinking [44]. For example, Xu et al. chemically grafted aminopropyl vinyl ether after activating the carboxyl groups of sodium alginate and obtainedalginic acid functionalized with vinyl ethers (Figure 1a) [45]. Photoinitiators can affect the crosslinking of hydrogels under UV irradiation. Bukhair et al. obtained cinnamoyl-modified photocrosslinkable alginic acid via multistep modificationand demonstrated that these crosslinked alginate hydrogels have better mechanical properties and stabilities, as well as biocompatibility with physically crosslinked alginate hydrogels due to the formation of cyclobutane bridges connecting the alginate polysaccharide chains through the (2π + 2π) cycloaddition reaction of the inserted cinnamoyl moieties [46]. They have great potential in biomedical applications.

Hyaluronic acid is also a linear macromolecular mucopolysaccharide. It has unique viscoelasticity, excellent water retention, biocompatibility, and nonimmunogenicity. It also has important physiological and biological functions and is widely used in clinical procedures [47,48]. However, natural hyaluronic acid has poor stability, is sensitive to hyaluronidase, and lacks mechanical strength. Therefore, chemical modifications are needed to prevent degradation and improve its mechanical strength, and the modified groups mainly include carboxyl, hydroxyl, and the amino group exposed by deacetylation. Lee et al. used Pluronic F127 to modify hyaluronic acid. The resulting polymers exhibited thermosensitive sol–gel transition behaviors over the temperature range of 20–40 °C. After modifying the functional groups of N-(3-dimethylamino propyl)methacrylamide, the HA-F127 polymer was polymerized to form a stable photocrosslinked hydrogel (Figure 1b) [49]. Jenjob et al. prepared microspheres by photocrosslinking bisphosphonates (alendronate) with methyl methacrylate-modified hyaluronic acid. The adsorption efficiency of the microspheres for cationic bone morphogenetic protein 2 (BMP2) reached 91.0% [50].

Chitosan is formed by removing some acetyl groups from the natural polysaccharide chitin. Its chemical structure is similar to that of hyaluronic acid. It is the only cationic polymer in nature [53]. It shows good biodegradability, biocompatibility, bacteriostasis, and other functions. Chitosan is usually insoluble in water and alkali solutions and needs to be dissolved with acid, so chitosan solutions are usually acidic. In addition, the commonly used gelation method for chitosan involves use of aldehyde-containing molecules (such as glutaraldehyde) as crosslinking agents to react with the amino groups of chitosan to form a Schiff base, leading to gelation [34]. In biomedical applications, acidic materials cause irritation to skin or wounds, while residual crosslinking agents have strong biological toxicity. To overcome these shortcomings, Zhou et al. modified chitosan with ethylene groups; the material was dissolved in water and mixed with polyvinyl alcohol modified by methacrylic acid. Photocrosslinked hydrogels were formed after a photoinitiatorwas added [54]. Monier et al. modified chitosan molecules with cinnamyl chloride. The crosslinked chitosan hydrogel showed good stability (Figure 1c) [51].

Heparin is a mucopolysaccharide sulfate composed of glucosamine, L-ieduraldehyde glycoside, N-acetylglucosamine, and D-glucuronic acid. It is strongly acidic. It is a natural anticoagulant in animals. Heparin specifically binds to growth factors, so it has important value in growth-factor-delivery systems [55,56]. Based on this, Yoon et al. modified heparin with N-methylacrylamide hydrochloride and then crosslinked it with cryloyl-modified Pluronic F127 to obtain composite hydrogels for the controlled release of growth factors [57]. Jeong et al. successfully prepared electrospun fiber scaffolds by mixing methyl acrylate-modified alginate and methyl acrylate-modified heparin with polyoxyethylene (PEO) through electrospinning and photocrosslinking, and found that they were effective inregulating cell behavior (Figure 1d) [52].

**Figure 2:**
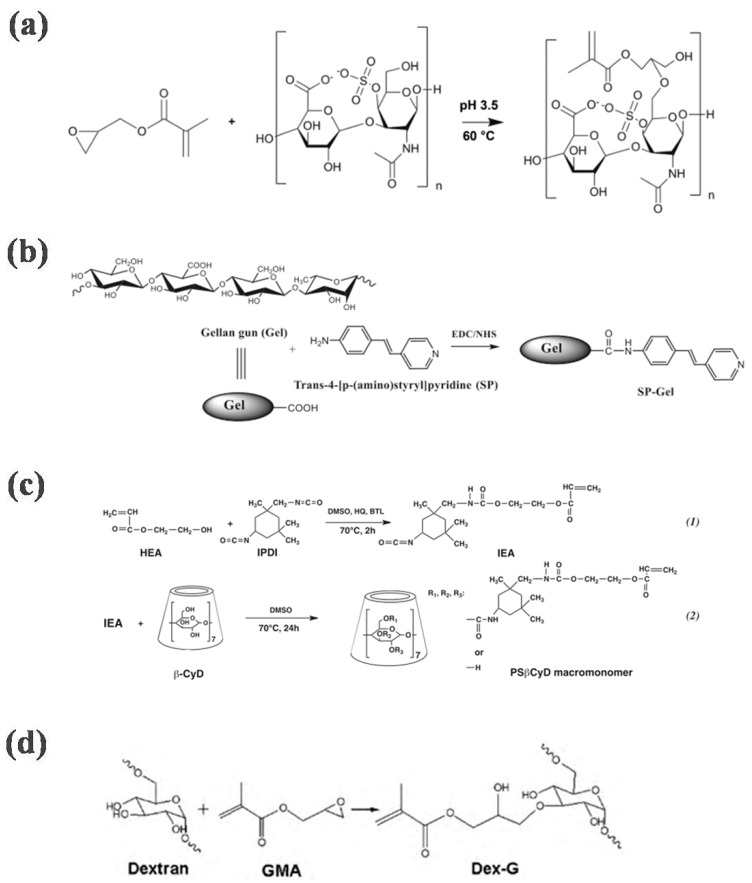
Synthetic routes for some crosslinked hydrogels. (**a**) Route map for the synthesis of chondroitin methacryloyl sulfate [62]. (**b**) Synthetic route for gellan gum modified by trans-4-aminophenyl pyridine [66]. (**c**) Synthetic route to cyclodextrin modified by isophorone diisocyanate and 2-hydroxyethyl acrylate [72]. (**d**) Synthetic route to glycidyl methacrylate modified dextran [73].

Chondroitin sulfate is a glycosaminoglycan covalently linked to proteins to form proteoglycans. It is widely distributed in the extracellular matrix and cell surfaces of animal tissues. The sugar chain is polymerized by alternating glucuronic acid and N-acetylgalactosamine disaccharides. It relieves pain and promotes cartilage regeneration [58]. After chemical modification, a chondroitin sulfate hydrogel can be obtained through photocrosslinking. The hydrogel has good biocompatibility and biodegradability, and has broad application prospects in drug delivery and bone repair [59,60]. For example, Kim and others prepared methacrylated PEGDA or chondroitin sulfate hydrogel and used it as a biomineralized three-dimensional scaffold for binding and deposition of charged ions [61]. Ornell et al. used the photocrosslinked methacryl group to covalently modify chondroitin sulfate and form injectable hydrogels, which can be used for sustained release of drugs over a certain period of time (Figure 2a) [62].

Gellan gum is a linear polysaccharide composed of glucose, glucuronic acid, and rhamnose. It is heat-resistant, acid-resistant, and enzyme-resistant and has good chemical stability. Gellan gum is insoluble in nonpolar organic solvents and cold water, but can be dissolved in hot water to form a transparent solution. After cooling, it becomes a transparent and solid gel [63,64,65]. Because of its good biocompatibility and tunability, researchers have tried to use gellan glue for tissue engineering. However, the gel formed by cations is hard and brittle, which limits its application. For this reason, a variety of photocrosslinking groups were used to modify gellan gum and obtain hydrogels with good biocompatibility and mechanical properties. For example, Oliveriraet al. modified gellan gum with trans-4-aminophenyl pyridine, and the product can be used for catalase immobilization after photocrosslinking (Figure 2b) [66]. Mano et al. modified gellan gum with methacrylic acid to prepare injectable gellan gum, which can be used as a self-generated osteogenic material [67].

Cyclodextrins are a series of cyclic oligosaccharides produced with amylose under the action of cyclodextrin glucosyltransferase. The three common cyclodextrins contain 6, 7, and 8 glucose units and arecalled α-, β-, and γ-cyclodextrins, respectively. These molecules are particularly attractive because they can form inclusion complexes with hydrophobic guests exhibiting appropriate molecular sizes and have high versatility. In addition, they can be chemically modified by hydroxyl substitution [68,69]. For example, Cosola et al. prepared a cyclodextrin modified with polyacrylate, which can prepare photocrosslinked materials with different morphologies via 3D technology [70]. Yamasaki et al. modified cyclodextrin with isophorone diisocyanate and 2-hydroxyethyl acrylate to obtain photocrosslinked cyclodextrin [71]. Microspheres of the prepared photocrosslinked polymer showed good separation efficiency for phenol (Figure 2c) [72].

Glucan is a homopolysaccharide formed with glycosidic linkages between glucose. Based on the types of glycosidic bonds, it can be divided into α-glucan and β-glucan. The common glucan is dextran, a common α-glucan. It has good biocompatibility and is widely used in the biomedical field [74]. Dextran is rich in active hydroxyl functional groups, so it can be chemically modified. Casadei et al. modified it with methacrylate to obtain photocrosslinked dextran, which enablesthe controllable release of polymers [75]. Yin et al. used glycidyl methacrylate to modify dextran and crosslinked it with methacrylic acid ethylene glycol-modified concanavalin A and polyethylene glycol dimethacrylateto obtain hydrogels (Figure 2d) [73]. The hydrogelsshowed good biocompatibility and glucose responsiveness and are expected to be used in glucose biosensors and intelligent insulin delivery.

### 3.2. Proteins/Peptides

Polypeptides are compounds composed of α-amino acids connected by peptide bonds. They are also intermediate products in protein hydrolysis. Amino acids form polypeptides via dehydration condensation. After winding and folding, polypeptides can form macromolecules with certain spatial structures, namely proteins. Proteins and peptides play important roles in the growth and development of life. Because of their widespread sources, nontoxic degradation products, and even benefits to the human body, proteins and polypeptide products are widely used in the biomedical field. Because the amino acids that make up proteins and peptides have free side-chain groups, they can be endowed with new functions through chemical modification, such as photocrosslinking groups. Common proteins used for modification with photocrosslinking groups include collagen, gelatin, keratin, silk fibroin, and albumin. Their application scope is greatly expanded through chemical modification, which gives them wide influence in the biomedical field.

**Figure 3:**
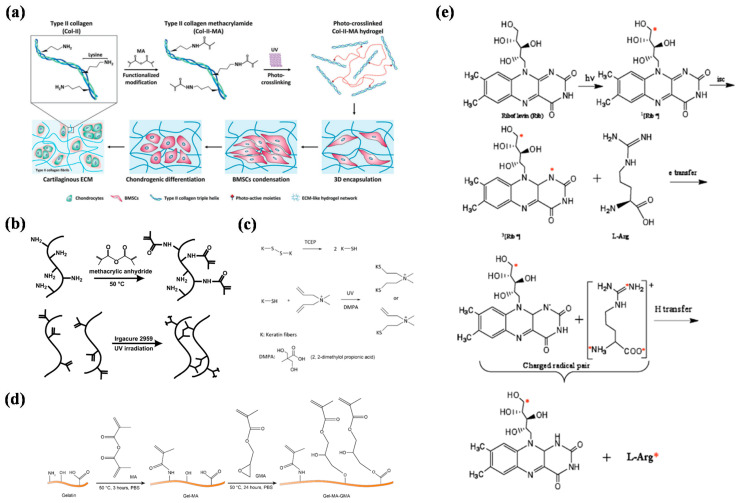
Part of the synthetic route maps or related schematic diagrams for crosslinked hydrogels. (**a**) Chemical modification of type II collagen and photocrosslinking hydrogel for bone-marrow mesenchymal stem-cell culture [79]. (**b**) Synthetic route of methacryloylated gelatin and its gelling diagram [80]. (**c**) Synthesis roadmap of two-step modified silk fibroin [81]. (**d**) Schematic diagram for crosslinking between keratin and 2,2-dimethoxy-2-phenylacetophenone [82]. (**e**) Schematic diagram of free-radical formation in the rib/L-Arg system [83].

Collagen is a protein formed by three peptide super-chain helices; it accounts for 25–30% of all proteins in mammals and is the most abundant protein [76,77]. Common collagens include type I, type II, type III, type V, and type XI. Collagen is widely used in foods, medicines, tissue engineering, cosmetics, and other fields because of its good biocompatibility, biodegradability, and biological activity [78]. Photocrosslinking of collagen hydrogel can be realized with chemical modifications and photocrosslinking. The degradation rate after the collapse of collagen hydrogels is slow, and they can be maintained for a long time to achieve specific functions. For example, Yang et al. photocrosslinked methacrylated type II collagen and used it for encapsulation of bone-marrow mesenchymal stem cells (BMSCs) (Figure 3a). The crosslinked collagen maintained its triple-helix structure, which provides a good microenvironment for proliferation and differentiationof BMSCs [79].

Gelatineis formed by partial hydrolysis of collagen, and it can be dissolved in hot water and forms a gel after cooling. This excellent feature provides flexible application scenarios. However, in most cases, a more stable hydrogel structure is desired, and the temperature range for gelatine gels is relatively narrow, which makes it difficult to meet the demand. Therefore, researchers have modified gelatinewith methacryloyl to enable photocrosslinking. Methacryloylated gelatine (GelMA) has good biocompatibility and structural stability after photocrosslinking. Therefore, methacryloylated gelatine shows great application value in the biomedical field [84]. The Khademhosseini team used gelatineas a raw material to synthesize GelMA, and used it in cell culture and microchips to verify its application value in cell-responsive microengineered hydrogels (Figure 3b) [80]. Fu et al. prepared structural colored microspheres with GelMA and used them for cell culture. They found that they had good biocompatibility and are expected to be used to construct liver chips [85].

Keratin is the main protein constituting hair, horn, claws, and the outer layers of animal skin. Because keratin contains cystine, it has a high proportion of disulfide bonds and plays acrosslinking role in protein peptide chains. Therefore, keratin has particularly stable chemical properties and high mechanical strength [86,87]. Studies have shown that keratin extracted from human hair fibers contains a leucine–aspartate–valine (LDV) cell-adhesion motif. Therefore, keratin has great application potential for cell culture and tissue engineering. To provide more application scenarios for keratin, it can be modified. For example, Yu and Hu et al. successfully realized photocrosslinking of keratin by using click chemistry and introducing 2,2-dimethoxy-2-phenylacetophenone (Figure 3c) [81,88].

Silk fibroin is a natural high-molecular fibrin extracted from silk. It has good mechanical and physicochemical properties and a long application history. To improve plasticity, many researchers have tried a variety of methods to modify it chemically (Figure 3d) [82]. Qi and others used silk fibroin modified by methacryl groups to obtain an injectable silk-fibroin hydrogel after photocrosslinking [89]. However, the lower side chains of silk fibroin decreased the content of methacryl groups, so the photocrosslinked silk-fibroin hydrogel had lower mechanical strength. Therefore, it was necessary to modify other active groups on silk fibroin to improve the mechanical strength. For example, Ju et al. obtained silk-fibroin hydrogels with good biocompatibility and mechanical properties by modifying the silk-fibroin hydroxyl with methacrylic acid and then performing crosslinking [90].

Albumin is a protein in plasma that maintains body nutrition and osmotic pressure, and it accounts for approximately 50% of all plasma proteins. It has good biocompatibility and solubility. Therefore, when preparing biomedical materials with albumin as a raw material, the inherent defects of synthetic materials can be avoided. For example, Chiriac and collaborators used riboflavin and arginine as natural initiators to crosslink bovine serum albumin (BSA) and obtain BSA hydrogels (Figure 3e) [83]. In vitro and in vivo experiments showed that the hydrogel had good biocompatibility.

## 4. Preparation of Photocrosslinked Hydrogels with Different Morphologies

In biomedical applications, different applications have different morphological requirements for common hydrogels. Therefore, it is crucial to prepare a hydrogel with a specific morphology. Common photocrosslinked hydrogel morphologies mainly include fibers, microspheres, thin films, microneedles, amorphous shapes, and so on. At present, many researchers have effectively explored processing methods for hydrogels with different morphologies, which has greatly expanded the application potential of hydrogels in biomedicine.

### 4.1. Fiber

**Figure 4:**
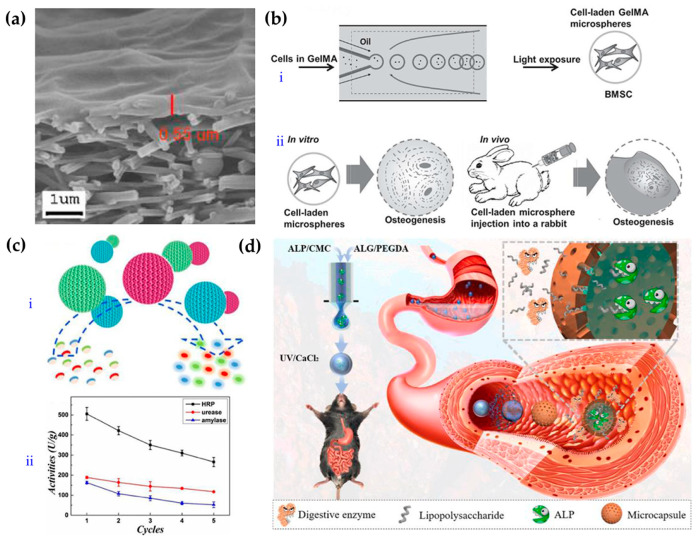
Schematic diagram of the preparation and application of hydrogels with fiber and microspherical morphologies. (**a**) Electron micrograph of ahigh-flux nanofiber-composite ultrafiltration membrane [98]. (**b**) Schematic diagram of the preparation and application of photocrosslinked GelMA-hydrogel microsphere [99]. (**c**) Enzyme-functionalized antiproteolytic-hydrogel microspheres were used for biocatalysis [100]. (**d**) Schematic diagram for preparation and application of ALP microcapsules [101].

Fibrous products such as gauze are widely used in the life science and biomedical fields. Inspired by this, many researchers have developed new hydrogel-fiber products. Due to their high surface-to-volume ratios and high porosities, they exhibit good water absorbance and air permeability and can replace some functions, such as those of traditional gauze in the biomedical field [91,92]. Usually, a hydrogel precursor solution is converted into fibers, photocrosslinked, and finally accumulated in woven-fiber products. At present, the main method used for fiber preparation is electrospinning [93,94,95]. When the solution is squeezed out under a high-pressure electric field, the liquid becomes filamentous due to the electric field. After the solvent evaporates, the polymer, polymer mixture, composite materials, and other molecules form fiber shapes. Electrospun fibers have many remarkable properties, such as high surface-to-volume ratios and functional tunability; they can be applicable in many areas including drug delivery, wound dressings, tissue engineering, membranes or filters, electronics, sensors, and energy [92]. Furthermore, when photocrosslinking technology is applied, the mechanical performance and stability of the fibercan be improved further [96,97]. For example, Tang et al. developed a novel high-throughput nanofiber-composite ultrafiltration membrane (Figure 4a) [98]. They first prepared chemically crosslinked polyvinyl-alcohol (PVA) nanofiberscaffolds on a nonwoven substrate, and then prepared a polyvinyl-alcohol (UV-PVA) barrier layer via UV crosslinking. The results showed that the 5 wt% UV-PVA solution coating provided an ultrafiltration membrane with high throughput and high retention rate after UV curing for 20 s. It had good pollution resistance and could be used for the separation of oil and water emulsions.

Microspherical hydrogels have excellent mobility and mass delivery, and thus have received considerable attention in fields such as biomedical detection and drug delivery. Specific structural differences can be used to subdivide the micropellets into homogeneous micropellets, antiopal micropellets [32], and nuclear-shell micropellets (microcapsules) [101].

### 4.2. Microballoons

#### 4.2.1. Homogeneous Micropellets

Homogenous hydrogel microspheres are usually formed by photocrosslinking after the hydrogel precursor solution is dispersed into liquid droplets. There are many ways to disperse solutions into droplets, but the principle is basically consistent; all methods use emulsification to generate droplets, such as with stirring, microfluidics, and electrospraying. Among these techniques, microfluidic technology has obvious advantages in preparing microspheres due to its accurate fluid control [99,102]. For example, Zhao et al. obtained hydrogel microspheres loaded with cells and growth factors by mixing cells and growth factors with GelMA solution, using microfluidics to cut them into droplets, and then photocrosslinking them. BMSCs in microspheres showed significant osteogenic effects both in vitro and in vivo, and significantly increased mineralization, thus promoting bone regeneration (Figure 4b) [99]. Combining microfluidic technology with electrojet technology allowed the application of an electric field as an additional shear force to simplify the microfluidic device. For example, Zhang et al. successfully used microfluidic electroinjection technology to prepare photocrosslinked chondroitin-sulfate microspheres, which showed good value for drug loading and wound repair [103].

#### 4.2.2. Antiopal Micropellets

Opal has a periodic, ordered structure formed by nanoparticles. When the nanoparticles have a specific size, the opal reflects light of a specific wavelength and thus shows a structural color. Negative replication using opal as a template yields an antiopal material. This material has the same structural color characteristics and a continuous porous structure, which provide good prospects for applications in mass transfer (especially in the fields of drug loading and sensing) [100]. For example, Wang and others prepared inverse-opal hydrogel microspheres by using colloidal-crystal microspheres assembled with silicon-dioxide nanoparticles as templates and AAm and N,N′-methylenebis-(acrylamide) (Bis) as photocrosslinked hydrogel skeletons to copy the template microspheres [32]. Afterhydrolysis and enzymatic immobilization of the microspheres, the resulting material had biocatalytic functionality and is expected to be used for treatment of complex water bodies (Figure 4c).

#### 4.2.3. Nuclear-Shell Microspheres (Microcapsules)

Antiopal hydrogel microspheres were obtained by using corrosion for removal of nanoparticle templates. If the corrosion was incomplete, the prepared microspheres had a nuclear-shell structure consisting of a hydrogel or nanoparticle-composite core and an antiopal hydrogel shell. These nuclear-shell microspheres exhibit responsiveness similar to that of antiopal hydrogel microspheres, and they also have high stability and can be used for encoding [104]. Based on this, Xu et al. developed photocrosslinked nuclear-shell hydrogel microspheres that can be used for miRNA detection by modifying the corresponding aptamer [105].

Homogeneous hydrogel microspheres are generally prepared by single-emulsion devices. Core-shell hydrogel microcapsules can be prepared if a double-emulsion device is used and the inner solute is not crosslinked. For example, Zhao et al. developed microcapsules containing an alkaline phosphatase (ALP) solution by applying microfluidic electrojet technology. The shells of the microcapsule were formed by calcium alginate and photocrosslinked PEGDA, which can preserve the activity of ALP in the digestive tract and thus be used for intestinal endotoxin cleaning (Figure 4d) [101].

### 4.3. Thin Film

**Figure 5:**
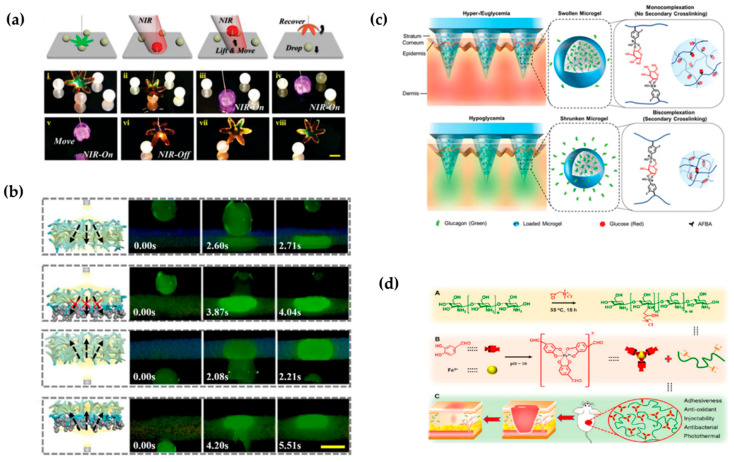
Schematic diagram for application of thin films, microneedles, and injectable morphologies of hydrogels. (**a**) A light-responsive antiopal hydrogel thin film captures and releases objects under light [109]. (**b**) One-way liquid discharge capacity of Janus sponge dressings [110]. (**c**) Schematic representation of the intelligent release of glucagon drugs contained by microneedles in vivo [111]. (**d**) Preparation principle for an injectable hydrogel with a double dynamic covalent bond and schematic diagram of its application in wound healing [112].

Among the many morphologies of photocrosslinked hydrogels, hydrogels with thin-film morphologies are most widely used because of their excellent size ranges and mechanical strength. Thin films are usually made of homogeneous structures, and by adding functional materials, hydrogel functionality can be enhanced in various ways. For example, incorporation of mesoporous active nanoparticles or a vascular endothelial growth factor can allow the hydrogel membrane to play an obvious promoting role in bone and wound healing [106,107]. Using the opal structure as the template, a hydrogel thin film with the antiopal structure was prepared. The nanoporosityand structure color of the antiopal structure enabled enhanced drug loading, sensing, and driving functions for hydrogels, withgood application prospects [37,108]. For example, Zhang et al. constructed photoresponsive antiopal hydrogel films that can be driven to capture and release objects under the action of light (Figure 5a) [109]. In some cases, two sides of the hydrogel film can be made to have different properties and functions by using different functionalization treatments to obtain a Janus structure thin film. Such films show good results when applied in complex environments. Zhang et al. prepared a Janus sponge dressing that was hydrophilic on one side and hydrophobic on the other. When the dressing was applied to the wound, the exudate was discharged from the wound to maintain the microenvironment of the wound, thus accelerating wound repair (Figure 5b) [110].

### 4.4. Microacupuncture Needle

Microneedles are usually arrays of microneedles, typically less than 1mm in length. Due to their size, they only penetrate the surface of the skin without causing bleeding; they cause much less pain than a traditional needle, and the trauma can heal in hours. Therefore, microneedles have obvious advantages in transdermal administration [113,114,115]. Compared with traditional metal- and silicon-based microneedles, hydrogel microneedles have good biocompatibility, large drug loads, and controllable drug release, and they have great development prospects. For example, Ghavaminejad et al. prepared microgel-loaded glucagon using phenylboric acid as a functional group, and then mixed the microgel with methacrylated hyaluronic acid (MeHA) and added it to the template [111]. After photocrosslinking, a photocrosslinked hydrogel microneedle was obtained. This microneedle can release glucagon to raise blood glucose under hypoglycemic conditions, thus preventing hypoglycemia after insulin injections for patients with diabetes (Figure 5c). The mechanical properties of the microneedles can also be controlled by adjusting the morphology. For example, Yu et al. designedan amifostine-loaded armored microneedle AAMN to havestronger mechanical properties than traditional conical microneedles, much higher mechanical strength than conical structuresand high skin permeability, and they can be better used for transdermal administration of amifostine [115].

### 4.5. Amorphous (Injectable) Hydrogels

Some hydrogels have poor mechanical properties due to limited high-molecular content or minimal crosslinking between large molecules. However, although these hydrogels do not maintain their specific fine structures and appearance, they can be used to cover irregular wounds and can be injected into irregularly shaped defect sites, which provides unique advantages in medical trauma repair. Therefore, such hydrogels have great application value in biomedical engineering. Liang et al. modified chitosan with quaternary ammonium moieties and formed an injectable hydrogel with trivalent iron, a protocatechuic aldehyde containing catechol and aldehyde groups. It can be used for wound healing and healing ofinfectionswith methicillin-resistant Staphylococcus aureus (Figure 5d) [112]. In addition, other functional materials can be added to injectable hydrogels to meet specific needs. For example, Zhao et al. combined drug-loaded hydrogel microspheres with injectable hydrogels to successfully prepare injectable hydrogels that were used for diabetes treatment [116].

## 5. Biomedical Applications

Photocrosslinked hydrogels are widely used in biomedicine, such as for biosensors, flexible wearable devices, medicine or tissue engineering, cell microcarriers, organ chips, and so on. It should be noted that when constructing hydrogels, appropriate components should be selected according to the application scenarios. For example, when a hydrogel is used for sensing, it should exhibit antiadhesion properties. When applied directly to humans, the hydrogel components should be biocompatible and preferably adaptable.

### 5.1. Biomedical Sensor

**Figure 6:**
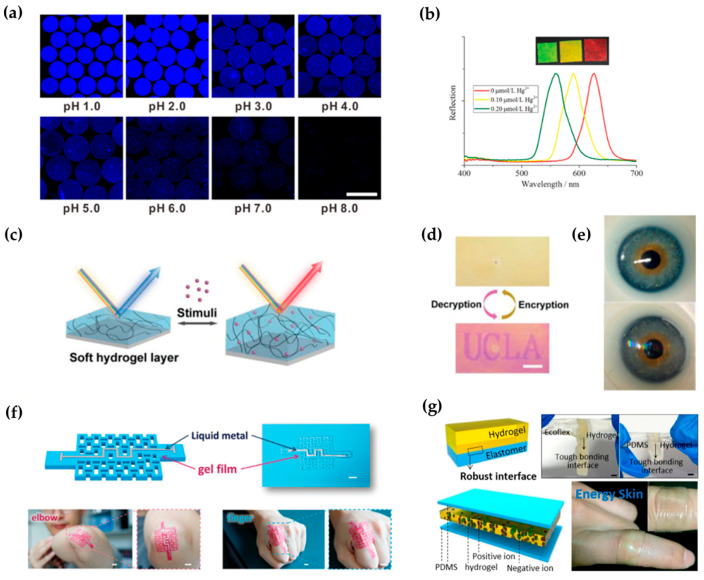
Applications of photocrosslinked hydrogels in biomedical sensors and flexible wearable devices. (**a**) The volume and fluorescence intensity of pH-responsive hydrogel microspheres changed under different pH conditions [117]. (**b**) The color of a mercury-ion-responsive hydrogels with structural color in mercury-ion solutions with different concentrations and reflection spectra of responses [118]. (**c**,**d**) Schematic diagram of hydrogel interferometer response to the target and demonstration of information encryption [119]. (**e**) Commercial contact lenses (top) and biosensing contact lenses (bottom) [120]. (**f**) Origami hydrogel for motion-signal sensing [121]. (**g**) Self-powered triboelectric nanogenerator that collects motion energy [122].

The components of hydrogels usually contain various functional groups, which can combine with molecules or ions in the surrounding environment and cause changes in the physical and chemical properties of hydrogels. By detecting these changes, the corresponding molecules or ions can be analyzed and sensed. For example, Qin et al. prepared hydrogel microspheres with a Janus structure, which changed its volume when the pH of the environment was changed; this led to changes in the intensitiesof surface-enhanced Raman scattering (SERS) signals and fluorescence signal. By collecting and analyzing these two signals, the pH of the solution could be determined (Figure 6a) [117].

When a hydrogel has an ordered micro/nanostructure, it can show structural color, which will change with volume changes of the hydrogel. Therefore, structural color can be used to detect and analyzethe target molecule. The Sun research group has performed a series of studies in this area. They used microspheres or membranes self-assembled with colloidal nanoparticles of silicon wafers as templates, used hydrogels for repeated preparation, and prepared a series of structurally colored hydrogel microspheres and films with different functions. These microspheres showed good sensing ability for detection of tumor markers, glycoproteins, DNA, and heavy-metal ions (Figure 6b) [118].

In addition to construction with micro/nanostructures, optical hydrogels can be directly prepared by using the principle of thin-film interference and the swelling characteristics of hydrogels. For example, Qin’s team proposed a hydrogel interferometercolor-change system that was simple to prepare, quick to respond, and easily patterned, and demonstrated various applications in detection and information encryption [119]. This kind of hydrogel does not need a fine micro/nanoconstruction unit, but is used to fabricate a film by rotating the coating on a highly reflective substrate with grafted functional groups. According to the principle of film interference, the hydrogel-film thickness is controlled to cause instant discoloration (Figure 6c). To achieve a portable detection device, researchers have also developed the necessary mobile phone software [119]. When an unknown sample must be analyzed, one takes a picture with the mobile phone, and the software automatically analyzes the subject and provides the test results. Reversible encryption and decryption can be realized according to whether the film is damp or not (Figure 6d) [119].

### 5.2. Flexible Wearable Devices

The concept of hydrogels was first proposed by Wichterle et al. in 1960. They constructed a three-dimensional hydrogel network comprising hydroxyethyl methacrylate (HEMA) containing a small amount of the crosslinking agent EGDMA to overcome the poor biocompatibility and stability of plastic products at that time. This hydrogel has a soft texture, good light permeability, adjustable mechanical properties and water content, and a certain degree of biological inertia. Based on these excellent properties, Wichterle used it forthe preparation of contact lenses [123]. Subsequently, many researchers made improvements on this material and developed cosmetic pupils with their own structural colors, which can be used for detection of substances in tear drops (Figure 6e) [120,124,125,126].

In addition to contact lenses, many flexible hydrogels are used in combination with sensing elements to produce flexible electronic devices that convert and conduct signals [127,128]. For example, Yu et al. developed a photocrosslinked acrylic hydrogel film with controllable thickness and excellent mechanical properties. During polymerization of the hydrogels, Zr^4+^ added to the solution coordinated with some of the carboxyl groups in polyacrylic acid, and the resulting hydrogels exhibited high stability. Akirigami structure for the hydrogel was obtained by photolithographic polymerization, so the hydrogel exhibited elevated ductility and flexibility to wrap the curved surface. After combining this origami hydrogel with a liquid metal, the resulting hydrogel sensor was used to sense arm or finger-bending motions (Figure 6f) [121]. To solve the need for a power supply for flexible electronic devices, Wang’s research group developed a stretchable triboelectricnanogenerator (TENG) based on elastomer hydrogels. The tensioning, transparent, ultrathin single-electrode TENG with a double-layer structure fit firmly to human skin and deformed as the human body moved. TENGs can also capture energy during deformation processes (pressing, stretching, bending, and twisting) to drive electronic devices (Figure 6g) [122].

### 5.3. Drug Delivery and Tissue Engineering

**Figure 7:**
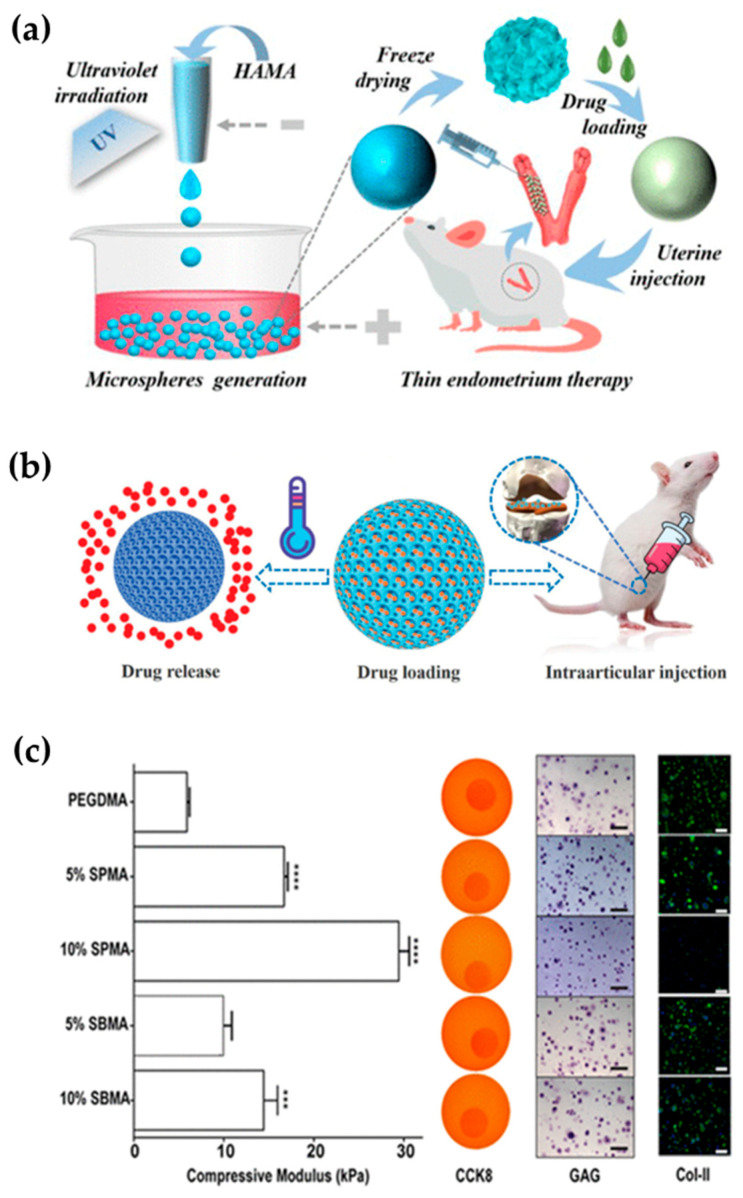
Application of photocrosslinked hydrogels in drug delivery, tissue engineering, and cell microcarriers. (**a**) Schematic diagram for the preparation of hydrogel microspheres for thin endometrium therapy [129]. (**b**) Application and principles of structural colored microspheres for treatment of osteoarthritis [132]. (**c**) Effects of different hydrogels on regulation of cell metabolism [133].

The traditional methods of drug administration such as injection and oral administration need to be given frequently, and will cause the problem of high drug concentration in a short time, resulting in side effects. Therefore, sustainable and low-dose drug delivery has important application prospects. The hydrogel material has a three-dimensional network structure that “locks” the drug in a grid, allowing it to be released slowly. As a result, hydrogels could be used for drug delivery, which in turn could play a role in tissue engineering. For example, Lei et al. prepared a photocrosslinked methacrylated hyaluronic acid (HAMA) microsphere loaded with vascular endothelial growth factor (VEGF) by a microfluidic electrospray technique and used in the treatment of thin endometrium. The combination of VEGF and HAMA can promote endometrial regeneration and embryo implantation, while hyaluronic-acid hydrogel scaffolds can work with VEGF to promote endometrial hyperplasia after degradation. Therefore, this drug-loaded hydrogel has a good application prospect (Figure 7a) [129,130,131].

By selecting suitable responsive hydrogels or changing the structure of nanopores, hydrogels can be designed to have tunable drug-delivery effects. The stimuli used to trigger the hydrogel’s response are usually light, heat, molecules, or ions. For example, Zhao et al. developed a glucose-responsive injectable hydrogel. Injected into the body, this hydrogel can maintain quasi-homeostasis in the normal range of blood glucose levels for approximately two weeks [116]. Salahuddin et al. prepared a light-controlled hydrogel. When exposed to light, the mesh of the hydrogel changes, altering the diffusion rate of the molecules being convenient for the controlled release of the drug [134]. Yang et al. prepared an antiopal microsphere and used its nanoporous structure to load drugs injected into the joint cavity. Elevated local temperatures during exercise or arthritis can promote the release of drugs, and vice versa. Therefore, this ingenious drug-delivery system could play an important role in the treatment of osteoarthritis (Figure 7b) [132].

### 5.4. Cellular Microcarrier

Photocrosslinked hydrogels can be used to load cells as microcarriers for cell culture, and biocompatibility and bioadhesion of hydrogels can be evaluated according to the growth status of the cells. For example, Liu et al. used microcarriers constructed from different hydrogels for cell culture, and found that the growth status of the cells with different microcarriers was inconsistent. This indicated that hydrogel-cell microcarriers can be used to study biological adhesion of different hydrogels [135].

When photocrosslinked hydrogels are used in cell culture, they can also be used as a platform to study cell growth, proliferation, and interaction between cells, which is also the preliminary basis for the application of hydrogels in tissue engineering in vivo. In earlier studies, Burdick’s research group prepared hydrogels with gradient-crosslinking density using microfluidic technology, which were used to study the migration and other behaviors of cells in vitro [136]. Dadsetan et al. obtained hydrogels with different crosslinking degrees and mechanical properties by changing the ratio of crosslinking agent and polymerizer in photocrosslinking hydrogels. When these hydrogels were used for chondrocyte culture, cells showed different adhesion effects and morphologies on hydrogels with different crosslinking densities [137]. Huang et al. found that by adding different ionic residues to the hydrogel, it can improve its mechanical properties and regulate the metabolic activity and collagen secretion of the loaded chondrocytes without changing the polymer content and swelling behavior (Figure 7c) [133]. These results indicated that hydrogels, when used as cell microcarriers, can simulate tissues in vitro and preliminarily monitor and regulate cell behavior [138].

### 5.5. Bionic Organ

**Figure 8:**
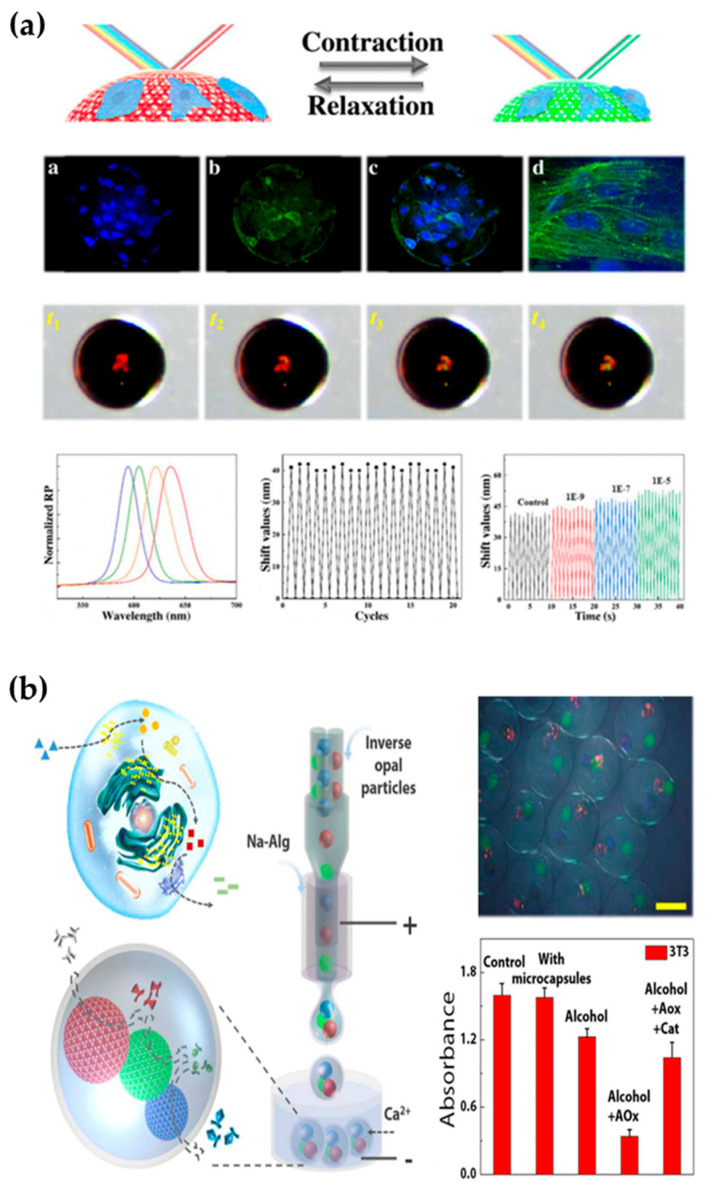
Application of photocrosslinked hydrogels in bionic organoids. (**a**) Bionic heart chip for cardiac drug evaluation [144]. (**b**) Bionic liver chip for the study of alcohol metabolism [145].

As mentioned earlier, photocrosslinked hydrogels can be used in cell culture. If microfluidic and other technologies are integrated into the cell-culture process, multiple cell cocultures can be realized, and the structures and functions of some organs can be simulated and realized through interactions between cells. The system is called an organ chip or organoid. These biomimetic organs can have important application value in the biomedical field. One of the most important applications is for drug evaluation. Today’s drug development process often requires multiple rounds of animal or human trials to verify the drug’s efficacy. Due to the differences between species, animal experiments often do not truly reflect the effects of drugs on humans, and both animal experiments and human experiments are faced with ethical problems. In addition, animal trials and human trials often take a long time, which greatly increases the cost of new drug development. Comparatively speaking, organ chips or organoids can prevent ethical problems and reduce time cycles, which is a good way to solve these problems. For example, Zhang et al. used a 3D microfluidic cell-culture system to construct microchips for liver, lung, kidney, and other organs for drug screening for related organ diseases [139]. Huh’s team constructed a biomimetic lung-chip microdevice, that simulated lung respiration by applying mechanical force, and this can be used to evaluate the biotoxicities of nanoparticles [140]. Furthermore, they conducted an evaluation study on the toxicities of drugs with lung microchips [141]. Toh et al. cocultured a variety of cells to construct a three-dimensional liver-organ chip. Studies have shown that this chip can simulate the function of the liver and be used for toxicity testing [142]. Zhao et al. combined structurally colored hydrogels with cardiomyocytes to construct a novel heart chip [143] and used optical signals to monitor cardiomyocyte activity and drug evaluation (Figure 8a) [144].

In addition to using bionic organs for drug evaluation, researchers have built organ chips for direct treatment of diseases. For example, Fu’s team used 3D printing technology to construct a bionic liver with microchannels and nanoparticles for metabolism and adsorption of toxic substances [85]. Wang et al. constructed a bionic enzyme-cascade microcapsule by using microfluidic electrojet technology and a structurally e-colored microsphere enzyme carrier. In such microcapsules, the cascade metabolism of alcohol in the liver can be simulated with an enzyme-cascade reaction (Figure 8b) [145].

## 6. Conclusions and Prospects

In general, synthetic photocrosslinked hydrogels are usually constructed by introducing molecules containing double bonds that can be polymerized by light at the end of the molecule, such as ethylene, acrylic acid, acrylamide, acrylic ester, etc. Natural-polymer photocrosslinked hydrogels are usually modified by acrylates or acrylamide derivatives, which are prepared by acylation of natural polymers containing reactive groups. In the presence of photoinitiator, photocrosslinked hydrogels can be carried out under mild conditions. Compared with other chemical crosslinking, photocrosslinked hydrogels can achieve in situ polymerization crosslinking and have the characteristics of fast reaction rate, mild reaction conditions, easy control of geometric shape, and low reaction heat release.

Photocrosslinked hydrogels are biomedical materials, and they have good development prospects. However, they still have some limitations. The synthetic monomers used to prepare photocrosslinked hydrogels are usually toxic. Removing these harmful substances and free-radical residues from photocrosslinked hydrogels is a challenging problem. Although chemically modified natural materials can vent monomer toxicity, their types are still limited, and the resulting mechanical properties are not as easy to control as those of hydrogels prepared from synthetic monomers. In addition, their high biocompatibility allows easy contamination with many bacteria, which limits their application in other fields. Therefore, in future research, more natural materials with chemical modifications designed to meet actual demand should be developed. In terms of preparation and application, although a variety of techniques have been used to prepare photocrosslinked hydrogels with different morphologies, it is still a great challenge to prepare photocrosslinked hydrogels that effectively simulate tissues or organs in vivo. In future research, the intersection of advanced processing technology, new materials and human-tissue mechanics will be important in solving this problem. We hope that with further combinations of biology, chemistry, engineering, and other disciplines, applications of photocrosslinked hydrogels in biomedical fields will be expanded and more remarkable achievements will be realized.

## Figures and Tables

**Table 1:** Four different photocrosslinked molecules and their molecular structures.

Functional Group Category	Single Structural Formula	Polymer Structural Formula
Vinyl	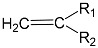	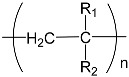
Acrylic class	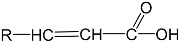	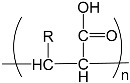
Acrylamide	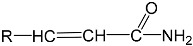	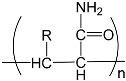
Acrylates	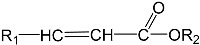	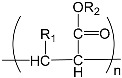

## Data Availability

Data are contained within the article.
